# Impact of HIV Infection and Kaposi Sarcoma on Human Herpesvirus-8 Mucosal Replication and Dissemination in Uganda

**DOI:** 10.1371/journal.pone.0004222

**Published:** 2009-01-20

**Authors:** Christine Johnston, Jackson Orem, Fred Okuku, Mary Kalinaki, Misty Saracino, Edward Katongole-Mbidde, Merle Sande, Allan Ronald, Keith McAdam, Meei-Li Huang, Linda Drolette, Stacy Selke, Anna Wald, Lawrence Corey, Corey Casper

**Affiliations:** 1 Department of Medicine, University of Washington, Seattle, Washington, United States of America; 2 Laboratory Medicine, University of Washington, Seattle, Washington, United States of America; 3 Deparment of Epidemiology, University of Washington, Seattle, Washington, United States of America; 4 Vaccine and Infectious Disease Institute, Fred Hutchinson Cancer Research Center, Seattle, Washington, United States of America; 5 Uganda Cancer Institute, Mulago Hospital, Makerere University, Kampala, Uganda; 6 Uganda Virus Research Institute, Entebbe, Uganda; 7 Infectious Diseases Institute, Mulago Hospital, Makerere University, Kampala, Uganda; 8 Academic Alliance for AIDS care in Africa, Kampala, Uganda; University of Sao Paulo, Brazil

## Abstract

**Introduction:**

Kaposi sarcoma (KS) is the leading cause of cancer in Uganda and occurs in people with and without HIV. Human herpesvirus-8 (HHV-8) replication is important both in transmission of HHV-8 and progression to KS. We characterized the sites and frequency of HHV-8 detection in Ugandans with and without HIV and KS.

**Methods:**

Participants were enrolled into one of four groups on the basis of HIV and KS status (HIV negative/KS negative, HIV positive/KS negative, HIV negative/KS positive, and HIV positive/KS positive). Participants collected oral swabs daily and clinicians collected oral swabs, anogenital swabs, and plasma samples weekly over 4 weeks. HHV-8 DNA at each site was quantified by polymerase chain reaction (PCR).

**Results:**

78 participants collected a total of 2063 orals swabs and 358 plasma samples. Of these, 428 (21%) oral swabs and 96 (27%) plasma samples had detectable HHV-8 DNA. HHV-8 was detected more frequently in both the oropharynx of persons with KS (24 (57%) of 42 persons with KS vs. 8 (22%) of 36 persons without, p = 0.002) and the peripheral blood (30 (71%) of 42 persons with KS vs. 8 (22%) of 36 persons without, p<0.001). In a multivariate model, HHV-8 viremia was more frequent among men (IRR = 3.3, 95% CI = 1.7–6.2, p<0.001), persons with KS (IRR = 3.9, 95% CI = 1.7–9.0, p = 0.001) and persons with HIV infection (IRR = 1.7, 95% CI = 1.0–2.7, p = 0.03). Importantly, oral HHV-8 detection predicted the subsequent HHV-8 viremia. HHV-8 viremia was significantly more common when HHV-8 DNA was detected from the oropharynx during the week prior than when oral HHV-8 was not detected (RR = 3.3, 95% CI = 1.8–5.9 p<0.001). Genital HHV-8 detection was rare (9 (3%) of 272 swabs).

**Conclusions:**

HHV-8 detection is frequent in the oropharynx and peripheral blood of Ugandans with endemic and epidemic KS. Replication at these sites is highly correlated, and viremia is increased in men and those with HIV. The high incidence of HHV-8 replication at multiple anatomic sites may be an important factor leading to and sustaining the high prevalence of KS in Uganda.

## Introduction

African (“endemic”) Kaposi sarcoma (KS) was originally described in Uganda in the 1960s [1]. Today, KS is one of the leading causes of cancer in Uganda, in children and adults of both genders [2], largely attributable to the dramatic increase in KS incidence in persons with HIV infection [3]. HIV associated (“epidemic”) KS has very high morbidity and mortality and has become a significant health problem in this region [4].

Human herpesvirus-8 (HHV-8) is the causative agent of all forms of KS [5], [6], and infection is endemic in Uganda, with an estimated seroprevalence between 36% and 60% [7], [8], [9], [10]. Despite this high seroprevalence, both endemic and epidemic KS occur in a minority of persons who are HHV-8 infected, and little is known about the underlying mechanisms determining the transition from asymptomatic HHV-8 infection to KS disease. Specifically, what role the site, persistence, and quantity of HHV-8 replication play in progression to KS is unclear. HHV-8 replication patterns, as measured by detection of viral DNA from samples collected from mucosal surfaces, have been defined in several longitudinal and cross sectional studies, with virus predominantly detected from the oral mucosa [11], [12], [13]. For example, a study of HHV-8 infected men in the US demonstrated HHV-8 by polymerase chain reaction (PCR) from 30% of oral swabs, 15% plasma and PBMC samples, and <1% of anogenital secretions [11]. Oral epithelial cells had detectable HHV-8 mRNA and DNA [11], and HHV-8 DNA in saliva was protected from digestion with DNase, consistent with the presence of virions [14].

Lytic HHV-8 replication in the vascular compartment is thought to play an essential role in the pathophysiology of epidemic KS [15]. Cross sectional studies of HHV-8 plasma viremia in patients with epidemic KS have shown an association with KS stage [16] and KS progression, but not KS burden [17]. The role of HHV-8 replication in non-epidemic forms of KS is less clear. HHV-8 viremia was not significantly associated with KS stage in classic KS [18], and HHV-8 viremia in persons with endemic KS has not been comprehensively studied. Whether loss of control of HHV-8 replication occurs in the oral compartment and precedes, occurs in conjunction with, or is independent of the development of KS is not known.

To gain insight into the pathophysiology of HHV-8 replication in association with endemic and epidemic KS in Uganda, we conducted a prospective cohort study using detailed sampling methods among HIV-infected and HIV-uninfected persons with or without KS to describe the sites and patterns of HHV-8 mucosal and plasma replication in a region where both HHV-8 and KS are highly prevalent.

## Results

### Study participants

**Figure 1:**
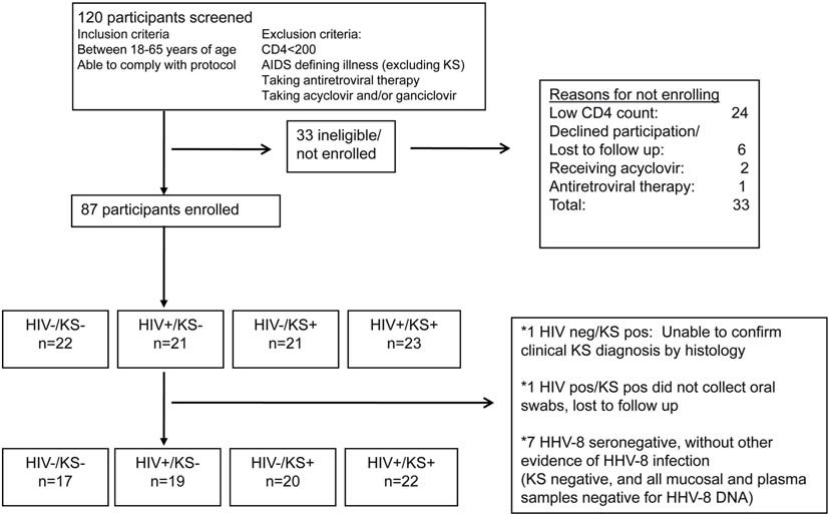
Enrollment and follow-up of participants in HHV-8 replication study.

Eighty-seven participants were enrolled in the study: one participant did not have histological confirmation of KS, and another participant enrolled but was lost to follow up before collecting any oral swabs: these participants were excluded from further analyses (Figure 1). Eighty-three (98%) of 85 participants had serum available for HHV-8 antibody testing, and of these 70 (84%) were HHV-8 seropositive. The two participants without serum available had biopsy-proven KS and therefore had evidence of HHV-8 infection. Similarly, 6 (46%) of 13 HHV-8 seronegative persons had clinical or virologic evidence of HHV-8 infection and hence had false negative HHV-8 serologies (3 persons had HHV-8 detected from ≥1 mucosal samples and biopsy proven KS, 1 had biopsy proven KS only, 2 had HHV-8 detected from mucosal samples on ≥1 occasion); these participants were therefore included in the analyses. Seven (54%) of 13 HHV-8 seronegative participants who did not have clinical or laboratory evidence of HHV-8 infection were excluded from further analyses.

The remaining cohort consisted of 78 participants who were distributed across four groups: 17 HIV negative/KS negative, 19 HIV positive/KS negative, 20 HIV negative/KS positive and 22 HIV positive/KS positive (Table 1). Eighteen (50%) of 36 participants without KS were women, compared with 10 (24%) of 42 participants with KS (p = 0.02). The median age was 32 (range 18–60) and did not differ across the groups (p = 0.50). There was no significant difference in proportion of members of the Baganda tribe (the most populous tribe in Kampala), in those with and without KS (p = 0.51). Among HIV-infected persons with and without KS, there was no difference in the median CD4+ T-cell count (p = 0.08), or median HIV plasma RNA level (p = 0.13). Among participants with KS, persons with HIV were more likely to have advanced disease, with presence of edema and/or visceral disease, including oral involvement [19] (p = 0.01). There were no differences between tobacco use (p = 0.06) or alcohol consumption (p = 0.88) in participants with and without KS. People with KS had a slightly later sexual debut compared to those without KS (19 years old vs. 18 years old, p = 0.03). Condom use was rare in all groups; HIV-infected persons had significantly more lifetime sexual partners compared to HIV-seronegative persons (p<0.001).

**Table 1:** Demographic, clinical and behavioral characteristics of participants.

	HIV−/KS−	HIV+/KS−	HIV−/KS+	HIV+/KS+
	N = 17	N = 19	N = 20	N = 22
**Female**	**11 (65)**	**7 (37)**	**5 (25)**	**5 (23)**
Median age (range)	34 (22–58)	32 (23–50)	32 (18–60)	35 (19–48)
Member of Baganda tribe	11 (65)	8(42)	6 (30)	13 (59)
Median CD4 count (number/mm^3^) (range) A	-	475 (309–829)	-	391 (208–1062)
HIV plasma RNA level, Log_10_ copies/ml (median, range) A		4.5 (2.3–5.6)		4.8 (2.3–5.9)
HHV-8 seropositive by IFA^B^	16 (94)	18 (95)	15 (75)	21 (96)
**Advanced KS** C, *	**-**	**-**	**6(30)**	**15 (68)**
Consume at least 2 alcoholic beverages per week	4 (24)	6 (32)	3 (15)	8 (36)
Smoker	3 (18)	6 (32)	-	4 (18)
Always uses condom with sexual activity^D^	1 (6)	6 (32)	6 (30)	3 (14)
**Median age at 1^st^ vaginal sex (range)**	**18 (12–25)**	**17 (8–25)**	**19 (11–28)**	**18 (15–25)**
Median number of vaginal sex partners in past yr (range)	1 (0–2)	1 (0–5)	1 (0–4)	1 (0–20)
**Median number of vaginal sex partners in lifetime (range)** *	**2 (1–7)**	**7 (1–80)**	**3 (0–15)**	**6 (2–50)**

All values are n (%) unless otherwise indicated.

Rows in bold indicate p-value<0.05, for comparison between KS positive and KS negative persons, unless otherwise stated. P-values were calculated using chi-square, Fisher's exact, or Kruskal-Wallis tests, when appropriate.

* p-value<0.05 for comparison between HIV positive and HIV negative persons, calculated using chi-square or Kruskal-Wallis test.

A Measured for HIV positive participants only.

B Data missing for 2 participants (1 HIV negative/KS positive, 1 HIV positive/KS positive).

C Only KS positive participants (n = 42) included in denominator.

D Data missing for 2 participants.

### HHV-8 detection from mucosal surfaces and plasma

**Figure 2:**
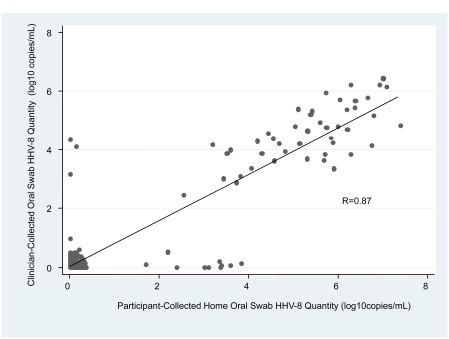
Correlation between HHV-8 detection in participant- and clinician- collected oral swabs. The quantity of HHV-8 DNA detected in oral swabs collected at home by participants was compared to those collected in clinic by trained clinicians. R = Spearman correlation coefficient.

Overall, 2969 (89%) of 3354 expected samples were collected and available for analysis, and subjects attended 377 (97%) of 390 protocol visits. From the 78 subjects, a total of 2063 oral swabs were collected on a median of 29 days per person and of these, 428 (21%) had HHV-8 DNA detected by PCR. The quantity of HHV-8 detected in oral swabs was highly correlated between participant-collected and clinician-collected oral swabs (Spearman correlation coefficient = 0.87, Figure 2). Among those oral swabs positive for HHV-8 DNA, the amount of HHV-8 detected from participant collected swabs was 0.9 log copies/ml higher than that detected from clinician collected swabs (p<0.001).

**Figure 3:**
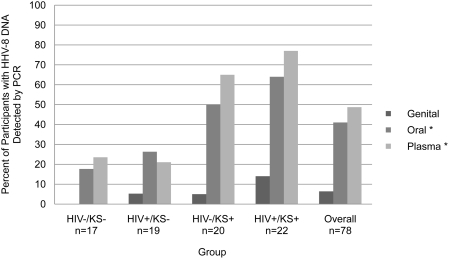
Proportion of participants with HHV-8 detected, by anatomic site and HIV and KS status. Percentage of participants with HHV-8 detected from oral swabs, genital swabs, or plasma samples. Participants collected a median of 29 oral swabs, 4 genital swabs, and 5 plasma samples. *p<0.05 for the comparison of the proportion of KS positive to KS negative participants with detectable HHV-8, using chi-square test.

Thirty-two (41%) participants had HHV-8 DNA detected from oral swabs (“shedding”) on ≥1 day. The proportion of participants with oral HHV-8 shedding was significantly higher in persons with KS as compared to those without KS (p = 0.002). Three (18%) HIV negative/KS negative persons and 5 (26%) HIV positive/KS negative persons had HHV-8 DNA detected from the oral mucosa. In contrast, 10 (50%) HIV negative/KS positive and 14 (64%) HIV positive/KS positive persons had oral HHV-8 shedding (Figure 3, Table 2). The median per person oral HHV-8 detection rate was highest in the persons with epidemic KS (27% of days), but was not significantly different in those with and without KS (p = 0.10) (Table 2). The median amount of HHV-8 detected from oral swabs was significantly lower in HIV positive/KS positive persons (3.9 log copies/ml), as compared to other groups (5.5 log copies/ml, p = 0.004).

**Table 2:** Frequency and Quantity of HHV-8 Detection among Participants with and without HIV and KS.

Oral Swabs	HIV−/KS−	HIV+/KS−	HIV−/KS+	HIV+/KS+	P value
	N = 17	N = 19	N = 20	N = 22	
Persons with HHV8 detected on ≥1 day, n (%)	3 (18)	5 (26)	10 (50)	14 (64)	**0.002** a
Per person HHV-8 detection rate, median (range)	0 (0–96%)	0 (0–100%)	2% (0–100%)	27% (0–100%)	0.10^b^
HHV-8 DNA log10 copies/mL*, median (range)	5.3 (2.7–6.9)	5.2 (2.8–7.3)	5.6 (2.3–7.8)	3.9 (2.7–6.1)	**0.004** c
**Plasma**					
Persons with HHV8 detected on ≥1 day, n (%)^e^	4 (24)	4 (21)	13 (65)	17 (77)	**<0.001** a
Per person HHV-8 detection rate, median (range)	0 (0–40%)	0 (0–100%)	20% (0–100%)	67% (0–100%)	**<0.001** b
HHV-8 DNA log10 copies/mL*, median (range)	2.6 (2.0–3.2)	3.3 (2.8–4.4)	2.9 (1.8–4.5)	3.4 (2.0–4.6)	**0.007** d
**Genital Swabs**					
Persons with HHV8 detected on ≥1 day, n (%)	0	1 (5)	1 (5)	3 (14)	0.36 a
Per person HHV-8 detection rate, median (range)^f^	0 (0–0)	0 (0–25%)	0 (0–33%)	0 (0–100%)	0.09 b
HHV-8 DNA log10 copies/mL*, median (range)	-	3.2^g^	4.2^g^	3.7 (3.2–4.6)	0.22^c^

* Among positive samples.

a P-value refers to comparison between KS negative and KS positive persons, using chi-square test.

b P-value refers to comparison between KS negative and KS positive persons, using GEE Poisson regression.

c P-value refers to comparison between HIV+/KS+ persons compared to the other groups, using GEE Gaussian regression.

d P-value refers to comparison between HIV positive and HIV negative persons, using GEE Poisson regression.

e Missing for 1 HIV+/KS+ participant.

f Missing for 2 HIV+/KS+, 2 KS+/HIV−, and 1 KS−/HIV− participants.

g Only 1 positive swab collected.

Three hundred fifty eight plasma samples were collected from the 78 participants (median of 5 separate samples per participant). Of these, 96 (27%) were positive for HHV-8 DNA. Thirty-eight (49%) participants had HHV-8 detectable in ≥1 plasma sample (Figure 3, Table 2). The proportion of participants with HHV-8 detected in plasma was significantly higher in persons with KS, as compared to those without KS (p<0.001). Among KS negative persons, 4 (22%) of 17 HIV-infected and 4 (21%) of 19 HIV-uninfected persons had detectable HHV-8 in the blood. Thirteen (65%) of 20 people with endemic KS and 17 (77%) of 22 people with epidemic KS had HHV-8 viremia. The median rate per person of HHV-8 viremia was 0% in persons without KS, 20% in persons with endemic KS, and 67% in persons with epidemic KS (p<0.001 for KS negative vs. KS positive). The median amount of HHV-8 DNA detected in plasma was significantly greater in participants with HIV (3.3 log copies/ml), as compared to those without HIV (2.8 log copies/ml), regardless of KS status (p = 0.007).

Of the 272 swabs collected from the genital tract, only 9 (3%) were positive for HHV-8 DNA and 5 (56%) of 9 came from one study subject who had genital KS. The median per person shedding rate and amount of genital HHV-8 DNA detected did not differ in KS positive and KS negative persons.

### Individual rates of HHV-8 detection, by HIV and KS status

**Figure 4:**
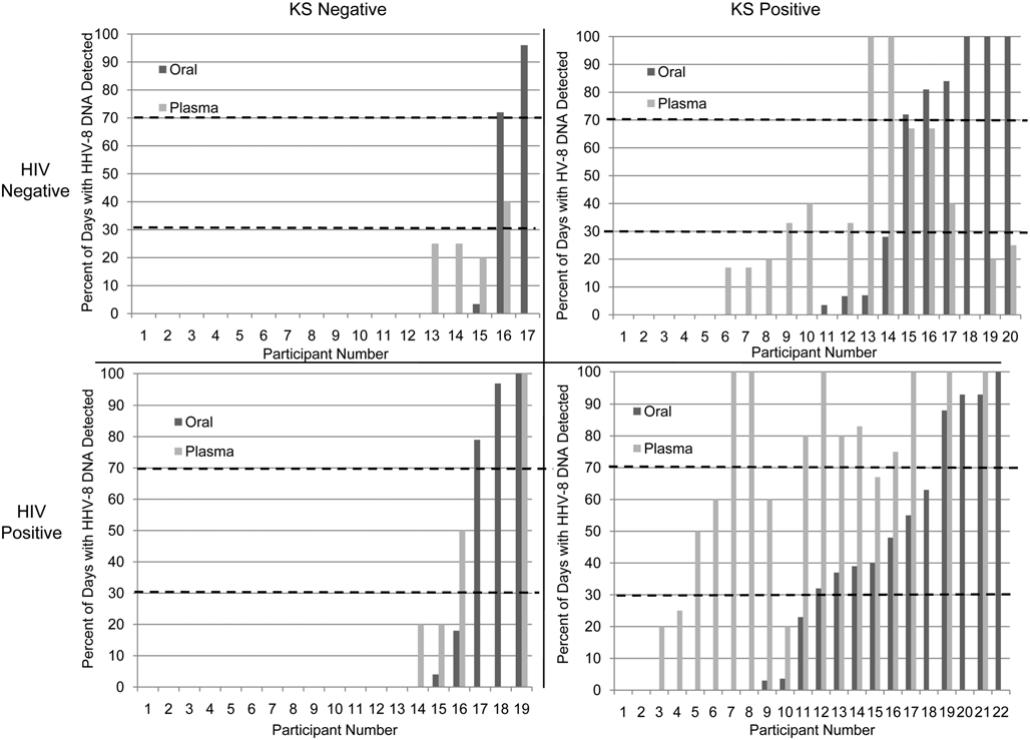
Rate of HHV-8 detection from oral swabs and plasma samples in individual participants, by HIV and KS status. Participants are numbered in ascending order according to oral shedding rate in each group. The participants collected a median of 29 oral swabs, 4 genital swabs, and 5 plasma samples per person.

Individual patterns of HHV-8 oral shedding differed by HIV and KS status. Interestingly, we identified several KS negative subjects who shed HHV-8 from their oropharynx at high frequency. HHV-8 was shed from the oral mucosa on greater than 70% of days in 2 of 3 HIV negative/KS negative and 3 of 5 HIV positive/KS negative participants who had oral HHV-8 detection (Figure 4). Six (60%) of 10 endemic KS participants with oral HHV-8 detected also shed HHV-8 from the mouth on greater than 70% of days. The other participants in these 3 groups exhibited low rates of oral HHV-8 detection (<30% of the days sampled). The pattern of HHV-8 oral detection was not dichotomous in epidemic KS, with 4 (29%) of 14 participants shedding on over 70% of days sampled, 7 (50%) participants shedding between 30% and 70% of days, and 3 (21%) shedding <30% of days.

In KS negative persons, HHV-8 viremia was more sporadic, present on less than 30% of days in 3 of 4 HIV negative/KS negative and 2 of 4 HIV positive/KS negative participants who had HHV-8 detected in the blood. Five (39%) of 13 participants with endemic KS were viremic on less than 30% of days, and two (15%) of 13 had HHV-8 viremia on 100% of days sampled. In contrast, ten (82%) of 17 participants with epidemic KS were viremic on greater than 70% of the days sampled, and 6 (35%) of these were viremic on 100% of days sampled.

We examined the association between HHV-8 oral shedding and viremia on a per person basis, and found that oral HHV-8 DNA predicted the detection of HHV-8 in the blood. HHV-8 was detected significantly more often from plasma samples collected when HHV-8 was shed from the oropharynx during the week prior than during periods when HHV-8 was not shed from the oropharynx (RR = 3.3, 95% CI = 1.8–5.9, p<0.001). Overall, 31 (62%) of 50 days with HHV-8 detected in plasma had HHV-8 oral shedding on at least one day during the prior week. Conversely, HHV-8 viremia did not predict oral HHV-8 detection in the following week (RR = 1.7, 95% CI = 0.8–3.6, p = 0.14).

### Correlates of HHV-8 oral shedding and viremia

Correlates of HHV-8 detection in the oral mucosa were determined by univariate and multivariate modeling (Table 3). In the univariate analysis, men had an increased risk of oral HHV-8 shedding (IRR = 3.1, 95% CI = 1.0–9.3, p = 0.05) and participants with KS trended toward an increased risk of oral shedding (IRR = 2.1, 95% CI = 0.9–5.0, p = 0.10). HIV infection did not increase the likelihood of oral shedding (IRR = 1.0, 95% CI = 0.5–2.2, p = 0.98), and HIV RNA level and CD4+ T-cell count were not associated with oral HHV-8 detection. In multivariate models, KS status and male sex remained associated with increased oral HHV-8 detection, but the association was no longer statistically significant. Having more than one sexual partner in the past year was associated with 3.4 fold increase in HHV-8 oral shedding rate (IRR = 3.4, 95% CI = 1.1–10.2, p = 0.03), while consistent condom use (IRR = 0.3, 95% CI = 0.1–0.9, p = 0.03), alcohol use (IRR = 0.4, 95% CI = 0.2–0.9, p = 0.02), and younger age (< = 15 years) at sexual debut (IRR = 0.2, 05% CI = 0.03–0.8, p = 0.02) were associated with a significantly reduced risk of oral HHV-8 detection (Table 3).

**Table 3:** Univariate and multivariate analyses of factors associated with HHV-8 oral shedding and viremia.

BIOLOGIC FACTORS	HHV-8 Oral Shedding				HHV-8 Viremia			
	Unadjusted		Adjusted		Unadjusted		Adjusted	
	IRR (95% CI)	P value	IRR (95% CI)^B^	P value	IRR (95% CI)	P value	IRR (95% CI)^B^	P value
Age	1.0 (1.0–1.0)	0.61	–		1.0 (1.0–1.0)	0.96	–	
Men (vs. women)	3.1 (1.0–9.3)	0.05	2.1 (0.6–7.4)	0.23	4.5 (2.0–10.1)	<0.001	3.3 (1.7–6.2)	<0.001
HIV seropositive	1.0 (0.5–2.2)	0.98	1.0 (0.5–2.0)	0.98	1.9 (1.0–3.4)	0.04	1.7 (1.0–2.7)	0.03
CD4 count^A^	1.0 (1.0–1.0)	0.71	–		1.0 (1.0–1.0)	0.62	–	
HIV plasma RNA level>4.5 log (vs.<4.5)^A^	1.2 (0.7–2.1)	0.62	–		1.6 (1.1–2.3)	0.02	–	
KS present	2.1 (0.9–5.0)	0.1	1.3 (0.6–2.8)	0.47	5.4 (2.3–12.5)	<0.001	3.9 (1.7–9.0)	0.001
Baganda tribe (vs. other tribes)	1.0 (0.5–2.1)	0.97	–		1.7 (0.9–3.1)	0.09	1.9 (1.2–3.1)	0.006
**BEHAVIORAL FACTORS**								
Alcohol use (vs. no alcohol use)	0.6 (0.3–1.4)	0.24	0.4 (0.2–0.9)	0.02	0.7 (0.4–1.4)	0.31	–	
≥1 sexual partner during past year (vs. no sex partners)	2.4 (0.9–6.8)	0.09	3.4 (1.1–10.2)	0.03	0.8 (0.4–1.4)	0.37	0.6 (0.4–0.9)	0.01
≤15 years of age at sexual debut (vs.>15 years)	0.1 (0.03–0.4)	0.001	0.2 (0.03–0.8)	0.02	0.5 (0.2–1.2)	0.14	–	
100% condom use (vs.<100% use)^C^	0.4 (0.1–1.5)	0.17	0.3 (0.1–0.9)	0.03	0.7 (0.3–1.6)	0.42	–	

Abbreviations: IRR = incidence rate ratio, CI = confidence interval, vs = versus.

A Calculated for HIV seropositive participants only.

B Multivariate model, adjusted for other variables indicated in table.

C Data missing for 2 participants.

In univariate analyses, correlates of HHV-8 plasma viremia included male sex (IRR = 4.5, 95% CI = 2.0–10.1, p<0.001), HIV infection (IRR = 1.9, 95% CI = 1.0–3.4, p = 0.04), plasma HIV RNA level above 4.5 log (IRR = 1.6, 95% CI = 1.1–2.3, p = 0.02) and having KS (IRR = 5.4, 95% CI = 2.3–12.5, p<0.001). In multivariate models, male sex, HIV infection, and KS status remained significantly associated with HHV-8 viremia. In addition, being a member of the Baganda tribe was associated with HHV-8 viremia (IRR = 1.9, 95% CI = 1.2–3.1, p = 0.006) and having more than one sex partner during the past year was associated with a reduced risk of viremia (IRR = 0.6, 95% CI = 0.4–0.9, p = 0.01). A sensitivity analysis, which included those participants without laboratory evidence of HHV-8 infection by either HHV-8 seropositivity, HHV-8 DNA detection from mucosal surfaces or blood, or biopsy proven KS, did not change the results of multivariate models for oral HHV-8 detection or viremia (data not shown).

## Discussion

We conducted a comprehensive study of the natural history of HHV-8 infection in an area where HHV-8 infection and KS are endemic and demonstrated; 1) HHV-8 DNA detection in the oropharynx and peripheral blood is common and highly correlated; 2) individual patterns of oral and plasma HHV-8 replication differ in persons with and without endemic and epidemic KS; and 3) HHV-8 viremia is more frequent among men and among persons with KS or HIV-infection.

While daily home collection of mucosal samples for herpesvirus culture and PCR has been validated in multiple studies in resource-rich settings, [11], [12], [20], [21], [22], [23], to our knowledge this is the first study to perform intensive mucosal sampling in a resource-limited setting. Our finding that the frequency of detection of HHV-8 DNA in oral swabs collected by the participant was highly correlated with that of clinician-collected swabs is an important operational research concept, for it demonstrates the feasibility of characterizing home-collected samples for DNA testing for infectious agents in field settings in resource-limited areas. Similar to results from shedding studies performed in the United States, participant collected swabs had significantly higher copy number of virus than clinician collected swabs [20], perhaps reflecting that once instructed on methods for sampling mucosal surfaces, self-obtained samples appear to be collected more consistently and vigorously than clinician-obtained samples.

We found that oral HHV-8 detection was associated with multiple recent sex partners, and that remote sexual debut and consistent condom use with sexual activity were associated with reduced risk of oral HHV-8 shedding. These results are consistent with potential acquisition of HHV-8 in adults, as previously described in a cohort of Kenyan men [24]. HHV-8 transmission in adults in this population could be through similar saliva sharing practices that put children at risk [25], [26], or it could be through other behavioral practices associated with high risk sexual activity, as seen with deep kissing in men who have sex with men (MSM) in the United States (US) [11], [27]. Elucidation of specific behaviors that are associated with transmission and oral replication of HHV-8 among Ugandan adults will require further study. Consistent with prior studies of HHV-8 replication patterns [11], [12], [28], HHV-8 was rarely detected from the genital mucosa in Ugandan men or women, particularly in the absence of KS.

HHV-8 viremia occurred frequently among persons with both epidemic KS and endemic KS. In addition, HHV-8 viremia appeared sustained throughout the 28 day study period in nearly 30% of persons with KS. While sustained and frequent viremia has been reported in patients with epidemic KS, our data in endemic KS provide further evidence that disseminated and persistent viral replication may be important to the pathophysiology of development of KS, independent of HIV status.

Our finding that HHV-8 viremia was associated with a greater than 3-fold increased risk of HHV-8 oral detection during the prior week suggests that a loss of control of HHV-8 replication in the mucosal compartments may contribute to the development of viremia and progression of KS. Our data are consistent with a model that lytic HHV-8 replication in the oral compartment precedes HHV-8 viremia, and that HHV-8 dissemination to the vascular compartment is necessary for development of KS. The high frequency of HHV-8 viremia in epidemic KS may also be a factor in sustaining and seeding areas of established KS [29], making eradication and therapy more difficult. These data indicate that understanding factors associated with HHV-8 replication in the mucosal compartment is important for understanding KS pathogenesis.

The observation of a dichotomous oral replication pattern in Ugandans with asymptomatic HHV-8 infection, between those with frequent and high quantity detection or infrequent and low quantity detection, is similar to results seen in a US based population of asymptomatic HHV-8 infected MSM [11], [12]. These data in combination with our observations that more frequent viremia is seen in men, especially members of the Baganda tribe, are intriguing aspects of the pathophysiology of KS in Uganda. Whether there are specific behavioral, environmental, or genetic cofactors that account for these observations is unclear. Familial clusters of classic KS have been described, without identified genetic predisposition [30], [31]. In French Guyana, the pattern of HHV-8 seropositivity was consistent with the presence of a major recessive gene predisposing to HHV-8 infection [32]. Polymorphisms in host IL-6 that result in increased IL-6 production were associated with KS development in HIV-infected men [33].

We were surprised that HIV infection did not have a more profound influence on HHV-8 oral replication and viremia in our cohort. In previous studies of women in Kenya, HIV seropositivity was associated with a 2-fold increased risk of HHV-8 detection from any mucosal surface [13]. In this study, HIV status, CD4+ T-cell count, and HIV plasma RNA level were not associated with oral HHV-8 detection. However, HHV-8 viremia was associated with HIV seropositivity in both univariate and multivariate analyses. It is possible that an association between HIV infection, HIV plasma RNA levels and HHV-8 oral shedding exists, but that our sample size was too small to detect it. Alternatively, our inclusion of only HIV-positive participants with CD4+ T-cell counts greater than 200/mm^3^ may have led to an underestimation of the relationship between HIV and HHV-8 replication, though previous work from our group and others has shown HHV-8 oral replication to be more common in HIV-positive persons with higher CD4+ T-cell counts [23], [34]. Interestingly, we found that despite more frequent HHV-8 detection in the oropharynx of persons with epidemic KS, the quantity of HHV-8 detected was lower compared with other groups. This has not been described in the only other study that compared HHV-8 oral replication patterns among HIV-infected persons with or without KS [35]. Careful studies of the biology of HHV-8 oropharyngeal replication may clarify the factors governing the frequency and quantity of HHV-8 oral shedding in saliva.

This study is limited by a short follow up period. Longer prospective studies of HIV-infected persons with high rates of oral HHV-8 detection and viremia are ongoing to determine if sustained HHV-8 replication is associated with the development of KS. If HHV-8 viremia does precede KS, as suggested by other cohorts [15], [16], [17], prevention of KS in HHV-8 viremic high risk populations with the use of antiviral agents with activity against HHV-8, such as valganciclovir [36],, would be of interest for further study.

In summary, we demonstrate that intensive virologic studies can be carried out in resource limited settings, and that lytic replication of HHV-8 in both the oral and vascular compartments is highly associated with the presence of endemic as well as epidemic KS. Detection of HHV-8 in these compartments is highly correlated, suggesting that mucosal HHV-8 replication may be an important antecedent of HHV-8 viremia. Understanding the cofactors that influence HHV-8 acquisition, reactivation and persistence is likely to lead to novel strategies to reduce HHV-8 associated KS.

## Methods

### Participants, Setting and Study Design

The study was conducted at the Uganda Cancer Institute (UCI), Mulago Hospital, Makerere University, Kampala, Uganda between May 2005 and July 2006. Participants between the ages of 18 and 65 were recruited from the UCI, the Infectious Disease Institute (IDI) and through word of mouth. Eighty-seven participants were enrolled into one of four groups on the basis of HIV and KS status: HIV negative/KS negative, HIV positive/KS negative, HIV negative/KS positive and HIV positive/KS positive. Participants unable to give written or verbal informed consent or taking acyclovir or ganciclovir were ineligible for the study. In addition, HIV positive participants who had a CD4+ T-cell count less than 200/mm^3^ or were taking antiretroviral medication (ARV) were ineligible. All participants gave informed consent for study participation and, when performed, HIV testing. Verbal consent was obtained in place of written consent if participants were unable to read and understand the consent form. Ethical approval for all study procedures, including verbal and written informed consenting procedures, was obtained from the Makerere University Research and Ethics Committee, the Uganda National Council for Science and Technology, and the University of Washington Human Subjects Division.

### Data and Sample Collection

At the screening visit, a questionnaire which collected information about demographic, sexual, and behavioral practices was administered. At enrollment, past medical history, current symptoms and medications were obtained, and a physical examination was performed, including detailed documentation of location and size of KS lesions. All participants with KS had histologically-confirmed disease. Participants were taught oral swab collection techniques, which included vigorously swabbing the buccal mucosa, gums, tongue, hard palate and tonsils with a Dacron swab. The swab was placed in a vial containing 1 ml of filter sterilized 1× digestion buffer [37] and stored at room temperature. Participants collected daily swabs of the oral mucosa at home for 28 days. Swabs were returned at weekly clinical visits, where a focused physical exam was performed and oral and anogenital swabs and plasma samples were obtained by a clinician. All samples were collected in duplicate. Samples were processed and stored at the Makerere University-Johns Hopkins University (MUJHU) laboratory at −80°C. One set of samples was shipped to the University of Washington for HHV-8 PCR testing and HHV-8 serology.

### Laboratory Testing

For those who could not provide written documentation of HIV test results within the past 6 months, HIV serostatus was ascertained using the Determine HIV ½ lateral flow qualitative rapid immunoassay on whole blood (Inverness Medical Innovations Inc.) Positive results were confirmed with the Clearview Stat-Pak (Inverness Medical Innovations Inc). CD4+ T-cell count was determined in HIV-positive persons at the MUJHU laboratory using standard cell sorting techniques. HHV-8 serostatus was determined at the University of Washington, using an immunofluorescence assay (IFA) to detect serum antibodies to latent and lytic proteins as previously described [38], with the presence of either considered serologic evidence of HHV-8 infection. DNA was extracted from mucosal swabs and plasma [12] and HHV-8 DNA was measured quantitatively with a real-time fluorescent polymerase chain reaction (PCR) with primers to the *orf73* gene, with positive and negative controls as previously described [11], [12]. Mucosal samples with ≥150 copies and plasma samples with ≥50 copies HHV-8 DNA/ml were considered positive for HHV-8 [39].

### Statistical Analysis

Chi-square tests or non-parametric Kruskal-Wallis (KW) tests were used to assess the homogeneity of demographic and virologic characteristics according to KS and HIV status. Quantity of HHV-8 DNA (copies/ml) was log_10_ transformed for analysis. Detection of HHV-8 in participant- and clinician-collected oral samples was evaluated with a Spearman-rank correlation coefficient. Oral shedding rates were calculated using participant-collected daily oral swabs only, unless such swabs were unavailable on clinic visit days, in which case the results from clinician-collected oral swabs were used. Summary shedding rates for oral, anogenital and plasma samples were calculated per participant (persons with at least one sample with HHV-8 detected/total participants) and per sample (swabs or plasma samples with HHV-8 DNA detected/total samples collected).

All regression models used generalized estimating equations (GEE) with an exchangeable correlation structure to adjust for intra-individual correlation of repeated measures per person, and the Huber-White variance estimator. Two sided p-values≤0.05 were considered statistically significant.

The difference in HHV-8 DNA copy number among clinician- and participant-collected oral swabs and among groups was determined using GEE Gaussian regression with an identity link. Correlates of HHV-8 oral mucosal and plasma detection were identified via univariate and multivariate GEE Poisson regression. All covariates that were associated with HHV-8 detection at the p<0.1 and p<0.2 levels in univariate analyses were assessed as significant predictors or confounders of HHV-8 detection. Confounders were retained if they changed the beta coefficients of predictors by greater than 10%. The association between HHV-8 viremia and oral HHV-8 detection was assessed using GEE binomial regression with a log link. Statistical analyses were performed using Stata, version 10.0.

## References

[1] Lothe F (1963). Kaposi's Sarcoma in Uganda Africans.. Acta pathol Microbiol Scand Suppl.

[2] Wabinga HR, Parkin DM, Wabwire-Mangen F, Nambooze S (2000). Trends in cancer incidence in Kyadondo County, Uganda, 1960–1997.. Br J Cancer.

[3] Beral V (1991). The epidemiology of cancer in AIDS patients.. AIDS.

[4] Gondos A, Brenner H, Wabinga H, Parkin DM (2005). Cancer survival in Kampala, Uganda.. Br J Cancer.

[5] Huang YQ, Li JJ (1995). Human herpesvirus-like nucleic acid in various forms of Kaposi's sarcoma.. Lancet.

[6] Moore PS, Chang Y (1998). Kaposi's sarcoma (KS), KS-associated herpesvirus, and the criteria for causality in the age of molecular biology.. Am J Epidemiol.

[7] Mayama S, Cuevas LE, Sheldon J, Omar OH, Smith DH (1998). Prevalence and transmission of Kaposi's sarcoma-associated herpesvirus (human herpesvirus 8) in Ugandan children and adolescents.. Int J Cancer.

[8] Wawer MJM, Eng SMM, Serwadda DM, Sewankambo NKMS, Kiwanuka NMp (2001). Prevalence of Kaposi sarcoma-associated herpesvirus compared with selected sexually transmitted diseases in adolescents and young adults in rural Rakai District, Uganda.. Sex Transm Dis.

[9] Gao SJ, Kingsley L, Li M, Zheng W, Parravicini C (1996). KSHV antibodies among Americans, Italians and Ugandans with and without Kaposi's sarcoma.. Nat Med.

[10] Simpson GR, Schulz TF, Whitby D, Cook PM, Boshoff C (1996). Prevalence of Kaposi's sarcoma associated herpesvirus infection measured by antibodies to recombinant capsid protein and latent immunofluorescence antigen.. Lancet.

[11] Pauk J, Huang ML, Brodie SJ, Wald A, Koelle DM (2000). Mucosal Shedding of Human Herpesvirus 8 in Men.. N Engl J Med.

[12] Casper C, Krantz E, Selke S, Kuntz SR, Wang J (2007). Frequent and Asymptomatic Oropharyngeal Shedding of Human Herpesvirus 8 among Immunocompetent Men.. J Infect Dis.

[13] Taylor MM, Chohan B, Lavreys L, Hassan W, Huang ML (2004). Shedding of human herpesvirus 8 in oral and genital secretions from HIV-1-seropositive and -seronegative Kenyan women.. J Infect Dis.

[14] Vieira J, Huang ML, Koelle DM, Corey L (1997). Transmissible Kaposi's sarcoma-associated herpesvirus (human herpesvirus 8) in saliva of men with a history of Kaposi's sarcoma.. J Virol.

[15] Whitby D, Howard MR, Tenant-Flowers M, Brink NS, Copas A (1995). Detection of Kaposi sarcoma associated herpesvirus in peripheral blood of HIV-infected individuals and progression to Kaposi's sarcoma.. Lancet.

[16] Campbell TB, Borok M, White IE, Gudza I, Ndemera B (2003). Relationship of Kaposi sarcoma (KS)-associated herpesvirus viremia and KS disease in Zimbabwe.. Clin Infect Dis.

[17] Nsubuga MM, Biggar RJ, Combs S, Marshall V, Mbisa G (2008). Human herpesvirus 8 load and progression of AIDS-related Kaposi sarcoma lesions.. Cancer Letters.

[18] Guttman-Yassky E, Abada R, Kra-Oz Z, Sattinger J, Perelman A (2007). Relationship between human herpesvirus 8 loads and disease stage in classic Kaposi sarcoma patients.. Diagnostic Microbiology and Infectious Disease.

[19] Krown SE, Metroka C, Wernz JC (1989). Kaposi's sarcoma in the acquired immune deficiency syndrome: a proposal for uniform evaluation, response, and staging criteria. AIDS Clinical Trials Group Oncology Committee.. J Clin Oncol.

[20] Wald A, Zeh J, Selke S, Ashley RL, Corey L (1995). Virologic Characteristics of Subclinical and Symptomatic Genital Herpes Infections.. N Engl J Med.

[21] Wald A, Zeh J, Selke S, Warren T, Ashley R (2002). Genital shedding of herpes simplex virus among men.. J Infect Dis.

[22] Wald A, Zeh J, Selke S, Warren T, Ryncarz A (2000). Reactivation of genital herpes simplex virus type 2 infection in asymptomatic seropositive persons.. N Engl J Med.

[23] Casper C, Redman M, Huang ML, Pauk J, Lampinen TM (2004). HIV Infection and Human Herpesvirus-8 Oral Shedding Among Men Who Have Sex with Men.. J Acquir Immune Defic Syndr.

[24] Baeten JM, Chohan BH, Lavreys L, Rakwar JP, Ashley R (2002). Correlates of human herpesvirus 8 seropositivity among heterosexual men in Kenya.. Aids.

[25] Mbulaiteye S, Marshall V, Bagni RK, Wang CD, Mbisa G (2006). Molecular evidence for mother-to-child transmission of Kaposi sarcoma-associated herpesvirus in Uganda and K1 gene evolution within the host.. J Infect Dis.

[26] Plancoulaine S, Abel L, van Beveren M, Tregouet D-A, Joubert M (2000). Human herpesvirus 8 transmission from mother to child and between siblings in an endemic population.. The Lancet.

[27] Casper C, Wald A, Pauk J, Tabet SR, Corey L (2002). Correlates of prevalent and incident Kaposi's sarcoma-associated herpesvirus infection in men who have sex with men.. J Infect Dis.

[28] Lampinen TM, Kulasingam S, Min J, Borok M, Gwanzura L (2000). Detection of Kaposi's sarcoma-associated herpesvirus in oral and genital secretions of Zimbabwean women.. J Infect Dis.

[29] Grundhoff A, Ganem D (2004). Inefficient establishment of KSHV latency suggests an additional role for continued lytic replication in Kaposi sarcoma pathogenesis.. J Clin Invest.

[30] Guttman-Yassky E, Cohen A, Kra-Oz Z, Friedman-Birnbaum R, Sprecher E (2004). Familial clustering of classic Kaposi sarcoma.. J Infect Dis.

[31] Cottoni E, Masia IM, Masala MV, Mulargia M, Contu L (1996). Familial Kaposi's sarcoma: case reports and review of the literature.. Acta Derm Venereol.

[32] Plancoulaine SGA, van Beveren M, Tortevoye P, Abel L (2003). Evidence for a Recessive Major Gene Predisposing to Human Herpesvirus 8 (HHV-8) Infection in a Population in Which HHV-8 Is Endemic.. J Infect Dis.

[33] Foster CB, Lehrnbecher T, Samuels S, Stein S, Mol F (2000). An IL6 promoter polymorphism is associated with a lifetime risk of development of Kaposi sarcoma in men infected with human immunodeficiency virus.. Blood.

[34] Gandhi M, Koelle DM, Ameli N, Bacchetti P, Greenspan JS (2004). Prevalence of human herpesvirus-8 salivary shedding in HIV increases with CD4 count.. J Dent Res.

[35] Laney AS, Cannon MJ, Jaffe HW, Offermann MK, Ou CY (2007). Human herpesvirus 8 presence and viral load are associated with the progression of AIDS-associated Kaposi's sarcoma.. Aids.

[36] Casper C, Krantz EM, Corey L, Kuntz SR, Wang J (2008). Valganciclovir for suppression of human herpesvirus-8 replication: a randomized, double-blind, placebo-controlled, crossover trial.. J Infect Dis.

[37] Ryncarz AJ, Goddard J, Wald A, Huang ML, Roizman B (1999). Development of a high-throughput quantitative assay for detecting herpes simplex virus DNA in clinical samples.. J Clin Microbiol.

[38] Chandran B, Smith MS, Koelle DM, Corey L, Horvat R (1998). Reactivities of human sera with human herpesvirus-8-infected BCBL-1 cells and identification of HHV-8-specific proteins and glycoproteins and the encoding cDNAs.. Virology.

[39] Magaret AS, Wald A, Huang M-L, Selke S, Corey L (2007). Optimizing PCR Positivity Criterion for Detection of Herpes Simplex Virus DNA on Skin and Mucosa.. J Clin Microbiol.

